# Role of Small GTPase RhoA in DNA Damage Response

**DOI:** 10.3390/biom11020212

**Published:** 2021-02-03

**Authors:** Chibin Cheng, Daniel Seen, Chunwen Zheng, Ruijie Zeng, Enmin Li

**Affiliations:** Department of Biochemistry and Molecular Biology, Shantou University Medical College, Shantou 515031, Guangdong, China; 17cbcheng@stu.edu.cn (C.C.); 18danielseen@stu.edu.cn (D.S.); delphinezheng@hotmail.com (C.Z.); ericrjzeng@hotmail.com (R.Z.)

**Keywords:** RhoA, DNA damage response, cell cycle arrest, DNA repair, Rac1, Net1, Ect2

## Abstract

Accumulating evidence has suggested a role of the small GTPase Ras homolog gene family member A (RhoA) in DNA damage response (DDR) in addition to its traditional function of regulating cell morphology. In DDR, 2 key components of DNA repair, ataxia telangiectasia-mutated (ATM) and flap structure-specific endonuclease 1 (FEN1), along with intracellular reactive oxygen species (ROS) have been shown to regulate RhoA activation. In addition, Rho-specific guanine exchange factors (GEFs), neuroepithelial transforming gene 1 (Net1) and epithelial cell transforming sequence 2 (Ect2), have specific functions in DDR, and they also participate in Ras-related C3 botulinum toxin substrate 1 (Rac1)/RhoA interaction, a process which is largely unappreciated yet possibly of significance in DDR. Downstream of RhoA, current evidence has highlighted its role in mediating cell cycle arrest, which is an important step in DNA repair. Unraveling the mechanism by which RhoA modulates DDR may provide more insight into DDR itself and may aid in the future development of cancer therapies.

## 1. Introduction

In research on signal transduction molecules, GTP-binding Rho proteins were identified as Ras homologs, and their biological functions were first scrutinized by experiments where Rho inhibition was induced by C3 exoenzyme and microinjection of Ras homolog gene family member A (RhoA) mutants. These functions mainly include stress fiber formation, focal adhesion formation, cytokinesis, and serum response factor (SRF)-dependent transcription. Downstream effectors of Rho, Rho-associated kinase (ROCK) and mammalian diaphanous-related formin (mDia), are thought to function in a collaborative way to control stress fiber formation and cellular contractility [1]. In detail, mDia as a formin promotes actin filament polymerization [2], and ROCK on the other hand increases myosin light chain (MLC) phosphorylation, producing contractile forces. In addition, ROCK phosphorylates and activates LIN-11, Isl1, and MEC-3 protein domain kinase (LIMK), which in turn phosphorylates and inactivates cofilin. Cofilin breaks down actin filaments, and therefore, the inhibition of cofilin leads to actin filament preservation [3]. Recent data suggest that these effectors may also participate in DNA damage response (DDR). The most extensively studied Rho proteins are RhoA, Ras-related C3 botulinum toxin substrate 1 (Rac1), and cell division control protein 42 (Cdc42) [4], which regulate cell motility via the control of lamellipodia and filopodia formation [5]. Rho GTPases cycle from GDP-bound status to GTP-bound status for their activation, and this process is regulated by guanine exchange factors (GEFs). Meanwhile, the hydrolysis of GTP to GDP is promoted by GTPase-activating factors (GAPs). Furthermore, lipid modifications of Rho (prenylation and palmitoylation) are essential for their membrane localization and functions, while guanine nucleotide dissociation inhibitors (GDIs) may sequester Rho in the cytoplasm [5].

Concurring with the role of RhoA in cytoskeletal regulation, it is important for the progression and metastasis of different human tumors including liver cancer and testicular cancer [6,7], colorectal cancer [8], hepatocellular carcinoma [9], osteosarcoma [10], and breast cancer cells [11]. However, recent studies have also suggested a tumor-suppressing role for RhoA in breast cancer [12] and colorectal cancer [13] in certain contexts. RhoA also regulates cell morphology during apoptosis. During the execution phase of apoptosis, the apoptotic microtubule network (AMN) adopts 2 different morphological patterns: round and irregular [14]. The former involves the neuroepithelial transforming gene 1 (Net1)/RhoA/ROCK1/MLC phosphorylation/actomyosin contraction signaling pathway that operates when apoptosis is induced by low concentrations of genotoxic drugs, promoting round-shaped apoptosis. In contrast, early caspase activation in response to high concentrations of genotoxic drugs—which induces Net1 and ROCK1 cleavage [15]—disrupts this signaling pathway and promotes irregular-shaped apoptosis. Furthermore, RhoA, which localizes at the cleavage furrow during telophase and ends up at the midbody at the abscission step, is involved in cytokinesis [16,17,18]. Interfering with RhoA activity by treating cells with Clostridium botulinum C3 exoenzyme or with RhoA expression by small interfering RNA (siRNA) silencing prevents contraction of the actomyosin ring and leads to failure of cytokinesis and multinucleation in various cell types [16,19].

DDR is a complex machinery that encompasses multiple pathways and enzymes acting to preserve genomic integrity. There are 3 steps included in this process: cell cycle arrest, DNA repair, and apoptosis if the damage is beyond repair [20]. In addition to such a plethora of research discussing the role of RhoA in cell morphology and motility regulation, there has been a rapid accumulation of data concerning a role of RhoA in DDR (for relevant details, see Section 5.2.1. “RhoA”). This particular role in DDR may contribute to the RhoA-related chemoresistance seen in multiple cancers [21]. In this review, we focus on the following 4 aspects of this topic: (1) how DNA damage induces RhoA activation; (2) Rho-specific GEFs Net1 and Ect2, and their roles in DDR; (3) the possible Rac1/RhoA interactions in DDR; and (4) RhoA-mediated cell cycle arrest and cell survival signaling.

## 2. RhoA Activation in Response to DNA Damage

RhoA functions as a signal transducer of DNA damage. In this section, we discuss the different pathways through which RhoA may be activated in DDR. In DNA double-strand break (DSB) repair, the lesion is sensed by the Mre-Rad50-Nbs1 (MRN) complex, which subsequently activates ataxia telangiectasia-mutated (ATM) kinase, which in turn initiates a flurry of enzymatic events including the checkpoint kinase 2 (CHK2)-mediated checkpoint. For single-strand break (SSB) repair, a lesion is sensed by the Rad9–Hus1–Rad1 complex, which activates ataxia telangiectasia and rad3-related (ATR) kinase and the subsequent CHK1-mediated checkpoint [20]. Some important data of this section are summarized in Table 1.

### 2.1. ATM and FEN1-Dependent RhoA Activation in DDR

Cytolethal distending toxins (CDTs) from Gram-negative bacteria are used to mimic the genotoxic effects rendered by ionizing radiation (IR), causing DSB formation, which triggers ATM activation, histone 2AX phosphorylation, MRN complex re-localization, CHK2, and p53 activation [22]. In addition to its activation downstream of CHK2 [20], p53 is also directly activated by ATM and ATR via phosphorylation on Ser15, as an initial step for cell cycle arrest during DDR [23].

In 2003, Frisan et al. reported that DSBs induced by Haemophilus ducreyi cytolethal distending toxin (HdCDT) triggers ATM-dependent activation of RhoA, which is not dependent on p53 activity. This study [24] first suggested the involvement of RhoA in DDR.

Flap structure-specific endonuclease 1 (FEN1) is a key enzyme in base excision repair (BER) and DNA replication [25,26], and it is related to the chemoresistance of multiple cancers such as breast cancer [27,28,29], non-small cell lung cancer (NSCLC) [30], and osteosarcoma [31]. In HeLa cells, FEN1 was found to promote RhoA activation after CDT intoxication, which is followed by p38 mitogen-activated protein kinase (MAPK) phosphorylation and further pro-survival signaling [32]. How FEN1 triggers RhoA activation is still obscure. One possible mechanism is that FEN1, in addition to its endonuclease activity, binds to ARHGDIA (a GDI), allowing RhoA activation during DDR, or it could be that FEN1 dephosphorylates Rho-specific GEF Net1 and activates RhoA indirectly [32].

### 2.2. Intracellular Reactive Oxygen Species (ROS)-Mediated RhoA Activation in DDR

ROS or oxidative stress may be generated by endogenous metabolism, inflammation, and exogenous oxidants and may induce a variety of DNA lesions [33]. Oxidative DNA damage contributes to aging [34] and multiple ailments such as male infertility [35,36,37] and psychiatric diseases [38,39].

RhoA activation is related to oxidative stress. When ROS/RNS presents in physiological levels with a high reduction potential, RhoA may be directly activated and may induce stress fiber formation. This activation is dependent on cysteines 16 and 20. However, when ROS/RNS is in excess, RhoA inactivation is expected [40]. ROS also preferentially targets DNA guanine [41], generating 8-oxo-7,8-dihydroguanine (8oxoG), which is removed by 8-Oxoguanine DNA glycosylase-1 (OGG1) during BER [42,43,44]. The resultant OGG1/8-oxoG complex functions as a GEF to activate RhoA and induces stress fiber formation [45]. The ROS-induced OGG1/8-oxoG complex also activates Ras and Rac1 [46,47,48]. In ROS-challenged mouse lungs, the OGG1/8-oxoG complex functions as a GEF to activate RhoA, which causes subsequent α-smooth muscle actin (SMA) polymerization into stress fibers, and this is proposed as a mechanism for how ROS-induced DNA damage leads to lung fibrosis and remodeling [45]. In terms of cell survival, ROS may inactivate certain phosphatases, preventing dephosphorylation of pro-survival molecules such as phosphorylated extracellular signal-regulated kinase (p-ERK) [49]. Conversely, ROS may also induce cell apoptosis in certain conditions, for example, in HCT116 cells, simvastatin leads to ROS generation and cell apoptosis [50].

**Table 1:** Studies elaborating the mechanisms of Ras homolog gene family member A (RhoA) activation in DNA damage response (DDR).

Mechanism	Cell Line	Genotoxic Agent	Reference
Ataxia telangiectasia-mutated (ATM)-dependent RhoA activation	HeLa, Human fibroblast	Haemophilus ducreyi cytolethal distending toxin (HdCDT), ionizing radiation (IR)	[24]
Flap structure-specific endonuclease 1 (FEN1)-dependent RhoA/p38 mitogen-activated protein kinase (MAPK) signaling	HeLa	Cytolethal distending toxin (CDT), IR	[32]
Reactive oxygen species (ROS)-mediated RhoA activation	REF-52 fibroblast, Rat2 fibroblast	Peroxide, Antimycin A ^1^	[40]
8-Oxoguanine DNA glycosylase-1/8-oxo-7,8-dihydroguanine (OGG1/8-oxoG) complex-mediated RhoA activation	MRC5 ^2^, MEF ^3^, Mouse lung extract	GOx ^4^, 8-oxoG	[45]

^1^ Antimycin A inhibits complex III in the electron transport chain, generating endogenous ROS. ^2^ Human diploid fibroblast. ^3^ Mouse embryonic fibroblast. ^4^ Glucose oxidase increases intracellular ROS.

## 3. Rho-Specific GEFs: Net1 and Ect2 in DDR

Neuroepithelial transforming gene 1 (Net1) and epithelial cell transforming sequence 2 (Ect2) are Rho-specific GEFs that preferentially localize in the nucleus [51,52,53] and play important roles in DDR. The breast cancer gene (BRCA)-1 C-terminal (BRCT) domain is highly conserved in checkpoint proteins [54], and Ect2 contains a tandem BRCT domain, making it a potential target for ATM. Loss of ATM inhibits Ect2 and Net1 activation after IR [55], which means that activation of the nuclear GEFs may depend, at least in part, on ATM function. In addition to Rho-dependent functions, current evidence has also suggested that Net1 and Ect2 have Rho-independent roles in DDR.

### 3.1. Net1 in DDR

Nuclear export of Net1 was regarded as a necessary step for activating Rho in the cytoplasm, and the pleckstrin homology (PH) domain of Net1 is thought to be essential for its nuclear export and subsequent activation [53]. More recently, Dubash et al. reported that, in the nucleus of multiple cell types, the majority of Net1 is active and a portion of the total RhoA reserve is actually located in the nucleus at steady state. This part of RhoA is regulated by the nuclear Net1 upon IR exposure, while the cytoplasmic part remains unaffected [56]. Two isoforms of Net1 have been identified: Net1 and Net1A. The longer isoform Net1 controls cell proliferation [57,58], and mitotic progression in that Net1 isoform is required for spindle assembly and the activation of p21-activated kinase 2 (PAK2) and Aurora kinase A independently of RhoA activation [59]. The other isoform, Net1A, modulates cell spreading on collagen, MLC phosphorylation, and focal adhesion maturation [60]. It was also found to regulate ATM activation and phosphorylation of H2A histone family member X (H2AX), which is also independent of RhoA [61]. Given the interaction between ATM and Net1A, it is reasonable to speculate that Net1A may be a mediator of RhoA activation downstream of ATM. Apart from that, mutant p53 in cancer cells may also stimulate RhoA/ROCK signaling through the upregulation of other GEFs [62].

After IR or CDT intoxication, Ser152 phosphorylation of Net1 is decreased [52], which is a hallmark for Net1 activation [63]. The mechanism of this dephosphorylation is unclear. PAK1, a downstream effector protein of Rac1, is known to phosphorylate Net1 isoforms on Ser152 and Ser153, inhibiting the GEFs’ function to activate RhoA [63]. However, the levels of Rac1 and PAK1 are not changed after IR or CDT intoxication, in HeLa cells or primary fibroblasts [56]. Interestingly, in HeLa cells exposed to supra-lethal doses (20 Gy) of IR, Net1-mediated RhoA activation is detected [52], while lower doses of IR do not promote RhoA activation [55]. This phenomenon suggests a possible threshold for DNA damage-induced RhoA activation. Contrary to the nuclear GEFs, the cytoplasmic GEFs (p115 RhoGEF, Vav2) are not activated after exposure to IR [55].

### 3.2. Ect2 in DDR

Ect2 plays a physiologic role in cytokinesis. Ect2 interacts with RhoA at the cleavage furrow to form a contractile ring for cleavage of the cell during telophase. The inhibition of Ect2 catalytic activity causes an accumulation of the GDP-bound inactive form of RhoA and results in cytokinesis failure and multinucleated cells [64]. Apart from Ect2’s physiologic role, it has been widely characterized to have oncogenic activities. It is found to be overexpressed in multiple types of human tumors [65]. In some cancers, Ect2 overexpression is driven by amplification of the ECT2 gene as part of the chromosome 3q26 amplicon, a common chromosomal mutation in human cancers [66]. In other tumors, including lung adenocarcinomas, Ect2 is overexpressed in the absence of gene amplification [67]. The overexpression of Ect2 is also associated with a poor prognosis in ovarian cancer patients [68].

Oncogenic Ect2 is often mis-localized to the cytoplasm to drive Rac1 activation, which results in cell transformation and growth [65,67]. Ect2 is also known to act from within the nucleus to activate nuclear Rac1 to drive the transformation of cancer cells [21]. In another model wherein Ect2 carries a N-terminal truncation, the Ect2 variant activates several Rho-mediated pathways, which promote cell transformation and survival [69].

Ect2 transcription is regulated by many factors. Scoumanne et al. found that Ect2 is downregulated in a p53-dependent manner in response to DNA damage [70]. While wild-type p53 inhibits the expression of Ect2, mutant p53 was found to increase Ect2, as observed in gastric cancer cells. This overexpression of Ect2 significantly promotes cancer cell proliferation, migration, and invasion [71]. Chen and colleagues also found that phosphorylation of residue Thr359 on Ect2 is essential for the oncogenic activity of Ect2. Elimination of the phosphorylation of Thr359 decreased RhoA activation and epithelial–mesenchymal transition (EMT), which resulted in the inhibition of the proliferation of malignant cancer cells [71].

Nuclear Ect2 was found to have tumor suppression activities in DDR. Soon after DNA damage, Ect2 was localized to the chromatin and DNA damage foci-like structures, where it aided in Ser15 phosphorylation of p53 and its activation and thereby regulated G1/S transition [72]. Ect2 is also required for apoptosis and activation of S and G2/M checkpoints in response to DNA damage [64]. It is not known how Ect2 is recruited to the chromatin and DNA damage foci-like structures and how it regulates p53 activation; however, it is proposed that the BRCT domain of Ect2 may interact with phospho-peptide sequences or BRCT domains of other foci proteins. Apart from the interaction of Ect2 with RhoA and Rac1, Ect2 is also involved in IR-induced RhoB-mediated cell death. Ect2 activates RhoB, which causes the downstream phosphorylation of c-Jun N-terminal kinase (JNK) and the induction of the pro-apoptotic protein Bcl-2 interacting mediator of cell death (Bim), leading to cell death [55].

## 4. Possible Rac1/RhoA Interaction in DDR

The Rac1-Topo IIβ axis strengthens the poisoning effect of anthracycline-topo II interaction, and Rac1 inhibition reduces the anthracycline-induced cytotoxicity by inhibition of the Rac1-Topo IIβ axis. Since this also occurs in the nucleus, Rac1 and RhoA may have extensive interactions during DDR, as proposed by Fritz and Kaina [73].

### 4.1. Rac1 in DDR

JNK and p38 MAPK are activated downstream of Rac1 as a part of a general stress response, and multiple transcription factors including activator protein-1 (AP-1), activating transcription factor 2 (ATF2), and nuclear factor kappa-B (NFκB) are mobilized to determine cell fate. JNK phosphorylates H2AX on Ser139 to initiate DNA repair processes. Non-receptor tyrosine kinase c-Abl is also regulated in a JNK/14-3-3 dependent manner, and it is believed to organize nuclear/non-nuclear communication under genotoxic stress, facilitating DNA repair. It has been shown that JNK activation is blocked by Rac1 inhibitors when cells are under genotoxic stress induced by ultraviolet-C (UVC) irradiation, alkylating agents, or methylating agents. (reviewed by Fritz et al. [74]). On the other hand, p38 forms a complex with MAPK-activated protein kinase 2 (MK2), which activates CHK1/2 and checkpoint mechanisms independently of ATM/ATR. Rac1 effector PAK1, found in the nucleus, induces cell apoptosis via PAK1/JNK1 signaling, and IR-induced nuclear PAK1 is also shown to modulate p53 activity [74]. PAK1 is also implicated in the activation of the microrchidia CW-type zinc finger 2 (MORC2) and Fanconi anemia (FA)/BRCA pathways, suggesting an important role of PAK1 in chromatin remodeling and DSB repair (reviewed in [75]). In further support of a pivotal role of Rac1 in DDR, the inhibition of Rac1 seems to attenuate the phosphorylation of player molecules in DDR such as p53, CHK2, and H2AX in different cell types and using different genotoxins. Moreover, Fritz et al. in their review [74] discussed the possibility that the nuclear actin structure controlled by Rho/Rac1 plays a critical role in DDR. However, there is also evidence that neither Rac1 nor Cdc42 is activated, and that only minimal levels of JNK activation are detected in HeLa cells after CDT intoxication or IR [52]. More recent studies [76,77] also showed that Rac1 activation is not involved in IR-induced DDR. This discrepancy of Rac1 activation could be related to different genotoxic agents applied in the studies, as proposed by Fritz and Kaina [73].

### 4.2. Rac1/RhoA Interaction

Many studies have suggested an inverse relationship between Rho and Rac [78,79,80], and this relationship is also closely related to ROS. The majority of intracellular ROS are from mitochondria [81], and non-mitochondrial ROS are generated by nicotinamide adenine dinucleotide phosphate hydrogen (NADPH) oxidase, which is regulated by Rac [82,83]. Rac1 activates the NADPH oxidase complex, generating ROS [84], which leads to redox-mediated inactivation of phosphatase low-molecular-weight protein tyrosine phosphatase (LMWPTP), elevation of p190RhoGAP activity, and subsequent inactivation of RhoA [85]. As mentioned earlier, Rac1 effector PAK1 may phosphorylate Net1 and several other GEFs to inhibit their function to activate RhoA. Conversely, active RhoA may inhibit Rac1 through the actions of ROCK (reviewed in [86]). Another evidence for this inverse relationship is that the inhibition of Rho/ROCK blocks the ability of sustained Ras-ERK/MAPK signaling to increase cyclin D1 transcription; however, this leads to rapid cyclin D1 induction downstream of Rac1 or Cdc42 [4]. Cdc42 is also upstream of p38 and JNK [87,88,89] as well as cyclin D1 activation [4].

**Figure 1:**
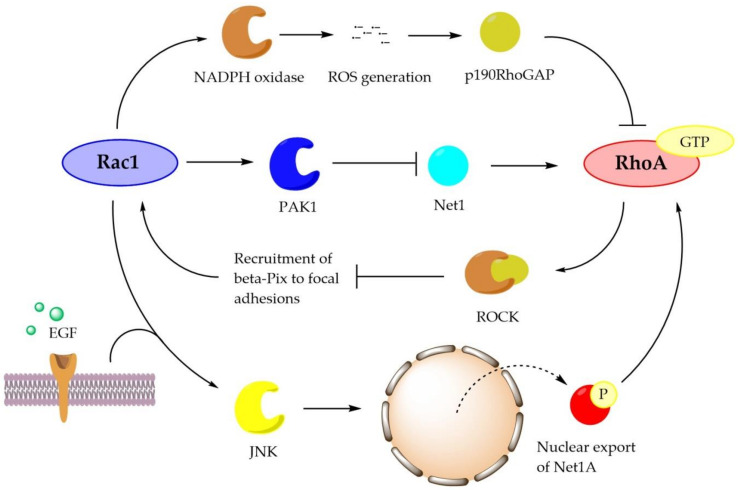
A brief summary of the Ras-related C3 botulinum toxin substrate (Rac1)/RhoA interaction: Rac1 may activate nicotinamide adenine dinucleotide phosphate hydrogen oxidase (NOX), which generates reactive oxygen species (ROS) and then promotes the activity of p190RhoGAP. The GTPase-activating factor (GAP) induces hydrolysis of GTP and inactivates RhoA. Rac1 may also downregulate RhoA through p21-activated kinase 1 (PAK1), which inhibits the guanine exchange factor (GEF) activity of neuroepithelial transforming gene 1 (Net1) and several other GEFs. Additionally, Rac1 may activate c-Jun N-terminal kinase (JNK), which phosphorylates Net1A and promotes its nuclear export. Net1A in the cytoplasm may promote RhoA activation. Conversely, active RhoA may inhibit Rac1 via actions of Rho-associated kinase (ROCK).

Adding more complexity to the Rac1/RhoA interaction, Rac1 may also promote RhoA activation. The nuclear export of Rho-specific GEF Net1 requires Rac1 activity [60], and more recent data provided by Ulu et al. [90] showed that cytosolic re-localization of Net1A downstream of epidermal growth factor (EGF) signaling [91] and integrin ligation [60] is entirely Rac1-dependent. They also demonstrated that, downstream of Rac1, 3 members of MAPKs, ERK, JNK, and p38, are involved in Net1A export and that activation of JNK or p38 is sufficient to trigger Net1A export even when EGF stimulation is absent. Net1A export is particularly sensitive to JNK, which phosphorylates Net1A on Ser52, preventing its re-import [90]. Nuclear exportin chromosome region maintenance 1 (CRM1) is also required for Net1A export, which is associated with the PH domain of Net1A [91]. Furthermore, Rac1 also stabilizes the cytoplasmic Net1A, which is independent of the PDZ domain containing protein Dlg1 that protects Net1A from proteasome degradation [92]. The Rac1/RhoA interaction described above is briefly summarized in Figure 1.

### 4.3. Rac1/RhoA Interaction during DDR May Be Worthy of Special Attention

In melanocytes treated with ROCK inhibitors, Rac1-mediated ROS initiates DDR, in which ATM is phosphorylated and p53 stabilized. p53 promotes the expression of P53-inducible gene 3 (PIG3), which is known to recruit the MRN complex to sites of DNA breaks [93]. PIG3 in turn increases ROS in the cytoplasm, and this further accumulation of ROS activates p190RhoGAP, leading to Rho/ROCK inhibition. In addition, Rac1 may promote PIG3 transcription in the long term [94]. Given the evidence so far, the intricate nature of Rac1/RhoA interaction beckons further elaboration of this topic under genotoxic stress.

## 5. Downstream Pathways of RhoA Activation in DDR

Current evidence has pointed towards RhoA’s function in cell cycle arrest during DDR. The roles of RhoA along with several other Rho members in cell survival signaling under genotoxic stress are also discussed in the following sections.

### 5.1. RhoA and Cell Cycle Arrest in DDR

#### 5.1.1. RhoA/p38 MAPK-Mediated Cell Cycle Arrest in DDR

There are 3 best-characterized MAPK (mitogen-activated protein kinase) pathways: ERK1/2 (mitogenic stimuli), JNK, and p38 (environmental stress and genotoxic stress) [95]. They are known to translocate from the cytosol to nucleus upon activation [90]. Among the MAPKs, p38 is known to be activated by genotoxic agents [95]. Focusing on RhoA, it may target different p38 MAPK isoforms depending on the stimuli. Downstream of RhoA, the p38γ MAPK isoform may activate c-Jun expression, inducing malignant transformation [96], while upon DNA damage, α- and β-p38 MAPK isoforms are activated [52]. p38 regulates a number of biological processes, among which cell cycle arrest is essential in DDR. p38 may induce G1/S arrest via regulation of cyclin D1; p53; retinoblastoma (Rb) phosphorylation; and the cyclin-dependent kinase (CDK) inhibitors p21, p27, and p57. p38 may also induce G2/M arrest via p53-dependent regulation of the cyclin-B/CDK1 complex and MK2-dependent Cdc25B inhibition (reviewed in [95]). In p53-deficient fibroblasts, ATM/ATR-induced activation of p38 MAPK/MK2 after cisplatin and doxorubicin treatment is recognized as the 3rd cell cycle checkpoint in addition to ATM/CHK2 and ATR/CHK1. Similar results were obtained in U2OS osteosarcoma cells where UV irradiation activates p38 MAPK/MK2 in an ATM/ATR-independent manner [97]. It was also demonstrated in HeLa and HCT116 cells that Net1/RhoA signaling is crucial for the activation of p38 MAPK/MK2 in response to CDT/IR stimuli, independently of ROCK [52]. MK2 is critical for the establishment of G2/M and G1/S checkpoints in p53-deficient cells, since it regulates the 14-3-3 binding of Cdc25B and the phosphorylation of Cdc25A [97].

#### 5.1.2. Rho/ROCK-Mediated Cell Cycle Arrest in DDR

ROCKs contribute to cell cycle progression. Deletion of ROCK1 and 2 results in severe cell proliferative defects, which is due to G1 arrest and block in cytokinesis. This does not involve p16, p21, or p27 but a downregulation of CDK1, cyclin A, and CDK inhibitor CKS1, and it is proposed that ROCKs control the levels of the 3 molecules by regulating actomyosin contractility [98]. The downregulation of ROCK also increases the level of another cell cycle inhibitor family, inhibitor of cyclin-dependent kinase 4 (INK4) [2], which binds to CDK4/6 and disrupts their interactions with cyclin D, inhibiting G1 progression [99].

ROCK1 may be activated by caspase-dependent cleavage; however, the cleaved ROCK1 is not detected in response to Adriamycin (doxorubicin) in MCF-7 cells [100]. ROCK2, possibly forming a complex with nucleophosmin (NPM) and BRCA2 at their centrosomes, is responsible for centrosomic duplication and therefore important for maintaining genomic stability [101]. ROCK2 is overexpressed in human hepatocellular carcinoma (HCC) cells, and its inhibition induces ubiquitin-mediated degradation of Cdc25A [102], which is responsible for cell cycle progression through the S phase via CDK2/Cyclin-E activation [20]. ROCK2 regulates Cdc25A by direct binding, and this mechanism is independent of CHK1/CHK2 [102].

However, how exactly ROCK2 may participate in DDR is still poorly understood. ROCK2 and LIMKs are downstream Rho partners in the nucleus [103,104]. Additionally, Rho/ROCK/LIMK signaling in response to genotoxic stress was established, in which RhoC and LIMK2 were identified as direct targets of p53. LIMK2 induced by genotoxic stress is essential for G1 arrest, and the inhibition of LIMK2 leads to prominent G2/M arrest and sensitization to DNA damage-induced apoptosis [100]. ROCK2 was also identified as a driver molecule of EMT in gemcitabine-resistant pancreatic cancer cells, acting through the ROCK2/p38/sp1/Zinc-finger-enhancer binding protein 1 (ZEB1) signaling pathway. ZEB1 induces chemoresistance via activation of the ATM/CHK1 mechanism [105], which may contribute to cell cycle arrest in DDR.

#### 5.1.3. RhoA Interacts with p21 (Waf1/Cip1) and p27 (Kip1) CDK Inhibitors and May Contribute to Cell Cycle Arrest in DDR

p53 acts downstream of ATM/ATR and is in charge of inducing G1 arrest via cyclin-E/CDK2 inhibition by p21 upregulation. Therefore, loss of p53 function, as observed in multiple tumors, leads to the dependence of intra S and G2/M checkpoint mechanisms during DDR [97]. p21 is also known to regulate S and G2/M arrests in DDR [99]. Sustained Ras-ERK/MAPK signaling triggered by mitogenic stimuli results in high levels of p21 and growth arrest, and this effect is counteracted by RhoA as it inhibits p21 transcription, promoting G1/S cell cycle progression [4].

In HeLa cells under gamma-irradiation, dominant-negative RhoA leads to prominent G1 and S arrests, while constitutively active RhoA preferentially induces S and G2/M arrests. This differentiation in cell cycle arrest may involve the dynamics of p21 [106]. However, in DDR without genetic manipulation, the ATM/ATR-mediated checkpoint mechanism overrides RhoA’s effect of G1/S progression [24]. In fact, overexpression of p21 is commonly seen in cancers and it may protect cancer cells from IR-induced apoptosis via inhibition of apoptotic CDKs. During DDR, p21 interacts with proliferating cell nuclear antigen (PCNA), preventing its function in DNA replication. However, when phosphorylated by activation of PI3K/protein kinase B (AKT) and retained in the cytoplasm, p21 dissociates from PCNA, promoting DNA synthesis and S progression, and disrupting DNA repair mechanisms. Cytoplasmic p21 may also bind to ROCK, suppressing Rho/ROCK signaling [99]. Therefore, in DDR, RhoA and p21 seem to work in concert with each other to achieve cell cycle arrest, despite the contrary, when a cell is under mitogenic stimuli. The p21 dynamics under UV irradiation is somewhat elusive, where p21 induction [107] and degradation [108] are both described.

On the other hand, nuclear p27 also inhibits the cyclin-E/CDK2 complex and thus inhibits G1 progression [109]. Phosphorylated p27 is also retained in the cytoplasm and promotes cell cycle progression via cyclin D1/CDK4 assembly and activation [110]. Mis-localized cytoplasmic p27, seen in cancers, selectively binds RhoA, inhibiting the activation of RhoA by the GEFs, and this promotes cell migration [111]. Conversely, RhoA is also known to downregulate p27 [4], possibly via mDia1, as shown in gastric cancer cells [112].

In A549 cells treated with doxorubicin, p27 is induced primarily by p38 MAPK, and inhibition of all of the phosphatidylinositol 3-kinase-related kinases (PIKKs) (ATM, ATR, and DNA-PKcs) significantly represses p27, implying the participation of p27 in DDR [113]. It is proposed that p21 and p27 may function as tumor suppressors in that they possess cell cycle arrest properties or as oncogenes when overexpressed in cancers in aid of DNA damage resistance [114]. However, how RhoA may interact with p21/p27 in the context of genotoxic stress still requires further elaboration.

#### 5.1.4. RhoA Modulates Cyclin D1 and May Contribute to Cell Cycle Arrest in DDR

During the late G1 phase of cell cycle and under mitogenic stimuli, cyclin D1 forms a complex with CDK4/6 and phosphorylates retinoblastoma (Rb) protein, which releases E2 promoter binding factor (E2F) family members. E2F factors promote the transcription of proteins necessary for S phase progression [4]. Cyclin D1 overexpression is in favor of chromosomal instability and cancer formation. Interestingly, it is also involved in DDR. Under a high dosage of IR, cyclin D1 degradation is a critical step for checkpoint establishment. However, a low dosage of IR is shown to promote the nuclear localization of cyclin D1, which activates Rad51—Cyclin D1 is recognized as a part of the Rad51-BRCA2 complex, which mediates homologous recombination repair (HRR) [115]. Additionally, cyclin D1 regulates estrogen- and androgen-mediated DDR [116].

Cyclin D1 level seems to be regulated by RhoA in a biphasic manner: cyclin D1 expression is blocked by RhoA at the early G1 phase and promoted by RhoA in concert with sustained ERK/MAPK activation at the mid-G1 phase [4,117]. However, how the cyclin D1 level is regulated by RhoA under genotoxic stress is still obscure. Another member of the Rho family, RhoE seems to regulate cyclin D1 level under genotoxic stress. After UVB irradiation, the level of cyclin D1 decreases [118,119], which is consistent with a report that RhoE is increased after UVB irradiation and that it blocks cyclin D1 posttranslationally [120]. Unexpectedly, however, RhoE knockdown induces prominent loss of cyclin D1, even in the absence of UVB [119], which suggests that a cyclin D1 maintaining role of RhoE may also exist.

### 5.2. Rho Family Members and Cell Survival Signaling in DDR

Thus far, 20 members of the Rho GTPase family have been identified, and they are classified into several subfamilies [1]. RhoA, RhoB, and RhoC, which form the Rho subfamily, and RhoE, which belongs to the Rnd subfamily, have certain implications in DDR and are discussed in the following sections.

#### 5.2.1. RhoA

In 1998, RhoA was found to protect HEp-2 cells from UVB-induced apoptosis via upregulation of B-cell lymphoma 2 (Bcl-2) and Bcl-X_L_ [121]. In osteosarcoma cells, RhoA was shown to activate the p42/p44 MAPKs/Bcl-2 survival pathway, which may be inhibited by lipophilic statins, inducing caspase-dependent apoptosis [122]. In HeLa cells, BRCA-1 interacting protein 1 (BRIP1) regulates the G2/M checkpoint in DDR, and BRIP1 overexpression suppresses RhoA, resulting in decreased cell survival [123]. In melanoma cell line MeWo, RhoA activity increases resistance to UV-induced DNA damage [124]. In the repair of DSBs, 2 distinct pathways are involved: homologous recombination repair (HRR) and non-homologous end joining (NHEJ) [20]. In HeLa cells under γ-radiation, RhoA inhibition by C3 transferase drastically reduces HRR and NHEJ, with H2AX phosphorylation unaffected [106]. The evidence mentioned above all suggest an essential role of RhoA in cell survival and imply its involvement in DDR.

#### 5.2.2. RhoB

RhoB is upregulated after UV irradiation, which is accomplished by p38 MAPK recruiting c-Jun and p300 to the RhoB promotor [125]. RhoB is also rapidly induced after DSB formation via mRNA stabilization by human antigen R (HuR), which is downstream of CHK2. Additionally, ATR/CHK1/smad ubiquitination regulatory factor 1 (Smurf1) signaling may inhibit RhoB degradation after DNA damage (reviewed in [126]). Downstream of Ect2/Net1-RhoB signaling in response to IR, the activation of JNK and the proapoptotic protein Bcl-2 interacting mediator of cell death (Bim) leads to apoptosis of breast and cervical cancer cells. In this study [55], RhoB knockdown does not affect the apoptotic machinery; therefore, it is proposed that RhoB acts as a signal amplifier when apoptosis is due. The proapoptotic Bim is also involved in apoptosis in a different manner. In HCT116 cells, simvastatin depletes the Geranylgeranyl pyrophosphate (GGPP) reserve of the cell and inhibits Rho protein prenylation, leading to the cytosolic re-localization of Rho proteins. Interestingly, the statin also increases the GTP loading of the unprenylated Rho GTPases (Rac1 and RhoA), which activate the NADPH oxidase (NOX) complex and generate ROS. This oxidative stress is known to activate JNK [127,128] and the downstream proapoptotic Bim to mediate apoptosis [50].

#### 5.2.3. RhoE

In keratinocytes, RhoE is proposed to regulate JNK, p38, and cyclin D1. After UVB irradiation, the level of RhoE is increased. At the same time, JNK and p38 are activated. RhoE knockdown causes the downregulation of p21, cyclooxygenase 2 (Cox-2), and cyclin D1, which are downstream of JNK and p38, mediating pro-survival signaling in keratinocytes [119]. RhoE inhibits Rho/ROCK signaling, blocking stress fiber formation. It is also considered a p53 target, promoting cell survival via the inhibition of ROCK1-mediated apoptosis. However in NHK and HaCat cells, despite the upregulation of RhoE upon UVB irradiation, its pro-survival effect is not dependent on p53 or ROCK1 [119]. A more recent study also demonstrated that genotoxic agents increase RhoE expression; however its stress fiber inhibition effect is overwhelmed by RhoC-mediated stress fiber formation [100].

## 6. Summary and Conclusions

**Figure 2:**
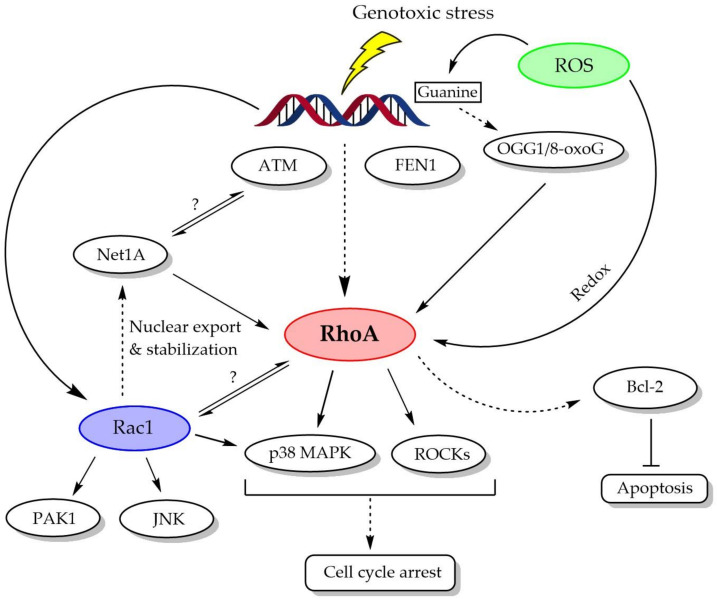
A brief schematic review of the role of RhoA in DDR: upon genotoxic stress, ataxia telangiectasia-mutated (ATM) and flap structure-specific endonuclease 1 (FEN1) are mobilized to activate RhoA, and this activation is also closely associated with ROS. In these processes, the participation of Rac1 and Rho-specific GEFs (especially, Net1A) may also be critical. Downstream of RhoA, mitogen-activated protein kinases (MAPKs) (especially, p38) and ROCKs seem to mediate cell arrest for DNA repair.

As illustrated in Figure 2, RhoA activation in DDR appears to be dependent on ATM and FEN1 activity, and intracellular ROS may also trigger RhoA activation directly (via redox reaction) and indirectly (via OGG1/8-oxoG complex). Interestingly, the Rho-specific GEFs Net1 and Ect2 are also found to function in DDR, with particular involvement of the Net1A isoform, which regulates ATM activation. In addition to being exported into the cytosol, nuclear Net1 activates nuclear RhoA, which may play an important role in DDR. On the other hand, Ect2 aids in p53 phosphorylation and checkpoint mechanisms in the early stage of DDR and is degraded in the late stage.

In many situations, there is an inverse relationship between Rac1 and RhoA, especially in the regulation of cell motility [80]. Rac1 may inhibit RhoA signaling via Net1 inhibition mediated by PAK1 or by manipulation of intracellular ROS. Rac1 may also increase RhoA activity by promoting Net1 export and its stabilization in the cytoplasm. Additionally, as reviewed by Fritz et al. [74], Rac1 may be an essential component of DDR in certain contexts. Therefore, further elaboration on the Rac1/RhoA interactions under genotoxic stress may bring more insights into the functions of the small GTPases in DDR.

In terms of cell cycle regulation, RhoA is expected to suppress CDK inhibitors p21 and p27, promoting G1/S cell cycle progression. However, this effect is invalid during DDR, resulting in G1/S arrest for DNA repair. Cyclin D1 is regulated by RhoA in a way that is in favor of G1/S progression, but their association under genotoxic stress still requires investigation. RhoE seems to negatively regulate cyclin D1 under UVB irradiation. p38 MAPK acts downstream of Rac/Rho and is induced by genotoxic stress, and it may mediate cell cycle arrest in the G1/S and G2/M phases via multiple pathways, as reviewed by Martinez et al. [95]. Among these pathways, Net1/RhoA/p38 MAPK/MK2 signaling is critical in the establishment of both G1/S and G2/M cell cycle arrests. Additionally, Rho/ROCK signaling may regulate cell cycle arrest via the regulation of actomyosin contractility. ROCK2, in particular, is essential for the stabilization of Cdc25A, promoting G1/S progression. However, under genotoxic stress, it may mediate cell cycle arrest.

Generally, several members of Rho family, RhoA, RhoB, RhoC, and RhoE, are upregulated in different cell lines and under different kinds of genotoxic stress. RhoA, RhoC, and RhoE appear to mediate cell survival signaling, while RhoB mediates apoptosis. In pro-survival signaling mediated by RhoA, ROCKs and p38 MAPK seem to play a critical role in cell cycle arrest induction, which allows for DNA repair. How Rac1 and cell cycle regulators (p21, p27, and cyclin D1) interact with RhoA in DDR still requires further exploration. Although evidence has made clear that RhoA is an indispensable component of DDR (at least for DSB repair), little is known about its interactions with the DNA repair mechanism itself. To further elaborate the role of RhoA in DDR, attention should also be drawn to the interactions between RhoA and participants of DNA damage repair in the context of genotoxic stress. In conclusion, RhoA is an essential component of DDR with a particular role of inducing cell cycle arrest. This adds to our understanding of the functions of RhoA and the detailed mechanisms of DDR, which may provide assistance in overcoming chemoresistance in cancer therapies.

## Abbreviations

ATM	Ataxia telangiectasia mutated
ATR	Ataxia telangiectasia and rad3-related
AMN	Apoptotic microtubule network
AP-1	Activator protein-1
ATF2	Activating Transcription factor 2
AKT	Activation of PI3K/protein kinase B
BER	Base excision repair
BRCA	Breast cancer gene
BRIP1	BRCA-1 interacting protein 1
BRCT	BRCA-1 C-terminal
Bcl-2	B-cell lymphoma 2
Bim	Bcl-2 interacting mediator of cell death
Cdc42	Cell division control protein 42
CHK1/2	Checkpoint kinase 1/2
CDT	Cytolethal distending toxin
CRM1	Chromosome region maintenance 1
CDK	Cyclin-dependent kinase
Cox-2	Cyclooxygenase 2
DDR	DNA damage response
DSB	DNA double-strand break
Ect2	Epithelial cell transforming sequence 2
ERK	Extracellular signal-regulated kinase
EGF	Epidermal growth factor
EMT	Epithelial–mesenchymal transition
E2F	E2 promoter binding factor
FEN1	Flap structure-specific endonuclease 1
FA	Fanconi anemia
GEF	Guanine exchange factor
GAP	GTPase-activating factor
GDI	Guanine nucleotide dissociation inhibitor
GGPP	Geranylgeranyl pyrophosphate
H2AX	H2A histone family member X
HRR	Homologous recombination repair
HuR	Human antigen R
IR	Ionizing radiation
INK4	Inhibitor of cyclin-dependent kinase 4
JNK	c-Jun N-terminal kinase
LIMK	LIN-11, Isl1, and MEC-3 protein domain kinase
LMWPTP	Low-molecular-weight protein tyrosine phosphatase
mDia	Mammalian diaphanous-related formin
MLC	Myosin light chain
MRN	Mre-Rad50-Nbs1
MAPK	Mitogen-activated protein kinase
MK2	MAPK-activated protein kinase 2
MORC2	Microrchidia CW-type zinc finger 2
Net1	Neuroepithelial transforming gene 1
NFκB	Nuclear factor kappa-B
NADPH	Nicotinamide adenine dinucleotide phosphate hydrogen
NPM	Nucleophosmin
NHEJ	Non-homologous end joining
NOX	NADPH oxidase
OGG1	8-Oxoguanine DNA glycosylase-1
8-oxoG	8-oxo-7,8-dihydroguanine
PAK1/2	P21-activated kinase 1/2
PIG3	P53-inducible gene 3
PCNA	Proliferating cell nuclear antigen
PI3K	Phosphatidylinositol 3-kinase
PIKK	Phosphatidylinositol 3-kinase-related kinase
RhoA	Ras homolog gene family member A
ROS/RNS	Reactive oxygen species/Reactive nitrogen species
Rac1	Ras-related C3 botulinum toxin substrate 1
ROCK	Rho-associated kinase
Rb	Retinoblastoma
SRF	Serum response factor
SSB	DNA single-strand break
SMA	α-smooth muscle actin
Smurf1	Smad ubiquitination regulatory factor 1
ZEB1	Zinc-finger-enhancer binding protein 1
